# Pan-cancer analysis of the immune aspects and prognostic value of NCAPG2

**DOI:** 10.1016/j.heliyon.2023.e18051

**Published:** 2023-07-13

**Authors:** Huidong Feng, Ning Chen, Huanhuan Li, Zhiwu Zheng, Hua Li, Haiyan Quan, Xing Jin, Ping Jiang, Qiong Wu, Xuejiao Yang

**Affiliations:** a Department of Infectious Diseases, Yanbian University Hospital, Yanji, Jilin Province, PR China; b Central Laboratory, Yanbian University Hospital, Yanji, Jilin Province, PR China

**Keywords:** NCAPG2, Cancer prognosis, Pan-cancer, Immune microenvironment, Immunotherapy

## Abstract

NCAPG2 has been reported to be associated with tumorigenesis in various types of cancer. However, data on the pathological mechanisms of NCAPG2 in pan-cancers remain lacking. Therefore, the study aimed to comprehensively elucidate the immune characteristics and prognostic of NCAPG2 in tumor microenvironments (TMEs). NCAPG2 was overexpressed in many tumor types, and this overexpression is related to poor clinical stages and prognosis. Furthermore, elevated NCAPG2 expression was strongly associated with TMEs. Moreover, gene set enrichment analysis was performed to investigate the pathways associated with NCAPG2, revealing its involvement in several immune-related pathways. Finally, we predicted the immunotherapeutic value and sensitivity to drugs based on NCAPG2 expression. Our study revealed that NCAPG2 could be utilized as an immune-related biomarker for both diagnosing and predicting the prognosis of multiple cancer types. Therefore, our findings suggest that targeting NCAPG2 in TMEs could be a promising therapeutic strategy.

## 1 Introduction

In recent decades, significant progress has been made in diagnostic methods and therapeutic strategies for cancer, resulting in improved quality of life and survival rates for patients. However, cancer continues to be a major contributor to illness and death worldwide, imposing a significant burden on both health and the economy [1]. TMEs have a pivotal role in the onset and progression of human cancers and are comprised of a variety of cells, of which a large proportion are infiltrating immune cells [2]. The mechanisms underlying the dynamic interplay between immune components and stromal of TMEs remain elusive. Tumor-immune cell interactions were first identified following the development of immunotherapies such as immune therapeutic vaccines and checkpoint blockade [3]. Immunotherapy has been shown to be effective in treating multiple types of cancer. Unlike traditional cancer treatments, immunotherapy works by reactivating the patient's immune systems [4]. At present, checkpoint-blocking drugs such as *anti*-CTLA-4, *anti*-PD-1, and *anti*-PD-L1 have been approved for cancer treatment [5]. Consequently, it is imperative to clarify the immunophenotypes and validate novel therapeutic targets in the context of cancer treatment.

Proper bi-oriented chromosome separation during mitosis is critically dependent on chromosome condensation [6]. The non-SMC condensin II complex subunit G2 (NCAPG2) protein is a part of the chromosome condensin II complex, which performs a critical function in mitosis, DNA repair, and histone modulation [7].

NCAPG2 has a regulatory role in carcinogenesis. For example, NCAPG2 regulates the G2/M phase to facilitate tumor proliferation, and its expression has been correlated with an unfavorable prognosis in lung cancer [8]. NCAPG2 overexpression also promotes hepatocellular carcinoma and malignant melanoma metastasis and proliferation through activating the NF-kB and STAT3 signaling pathways [9] and suppress apoptosis by activating the STAT3 signaling pathway [10]. Studies on colon cancer have demonstrated that NCAPG2 holds promise as both a therapeutic target and a biomarker for cancer prognosis and treatment [11]. Nevertheless, evaluation of NCAPG2 in previous studies remains limited to a few cancers; therefore, the clinical implications and biological functions of NCAPG2 in cancer as a whole are still unclear and require further clarification.

In this study, we aimed to systematically investigate the expression profile of NCAPG2 in multiple cancer types, using transcriptional and clinical data from public datasets. We also conducted analyses differences in mutations, protein levels, prognostic values, and biological functions of NCAPG2. We further examined the correlation between NCAPG2 and immune cell infiltration, microsatellite instability (MSI), tumor mutational burden (TMB), immune-related genes, and immune checkpoint gene in different TMEs. We also evaluated the immunotherapy value of NCAPG2 for various cancers using immunotherapy cohorts. Finally, we utilized public databases to predict the immunotherapy response and identify candidate small-molecule drugs that exhibit sensitivity across multiple cancer types, based on the expression of NCAPG2.

## 2 Materials and methods

### 2.1 Acquisition of data

The cell lines data were obtained from the CCLE dataset (https://sites.broadinstitute.org/ccle/). In addition, clinical and transcriptional data from The Cancer Genome Atlas (TCGA), Therapeutically Applicable Research to Generate Effective Treatments (TARGET), and Genotype-Tissue Expression (GTEx) datasets were downloaded from the UCSC Xena database (https://xenabrowser.net/). The cBioPortal database (https://www.cbioportal.org/) was utilized to analyze the frequency of alterations, mutation types, mutated sites, copy number alterations, and three-dimensional (3D) structure of NCAPG2.

### 2.2 Protein level analysis

To analyze protein expression in the Clinical Proteomic Tumor Analysis Consortium (CPTAC) dataset (https://cptac-data-portal.georgetown.edu/), the UALCAN database (http://ualcan.path.uab.edu/analysis.html) was employed.

### 2.3 Prognostic analysis

The connection between NCAPG2 and patient prognosis, including disease-specific survival (DSS), progression-free interval (PFI), disease-free interval (DFI), and overall survival (OS), was investigated using Kaplan-Meier (KM) curves and forest plots.

### 2.4 Immune infiltration and immune modulator gene analysis

For predicting the immune characteristics of NCAPG2, transcriptional data were evaluated through various algorithms such as ESTIMATE and immunedeconv package, which includes TIMER, CIBERSORT, MCP-counter, xCell, EPIC, and quanTIseq.

### 2.5 PPI network and enrichment analysis

The GeneMANIA database (http://www.genemania.org) was utilized to construct the protein-protein interaction (PPI) network of NCAPG2. NCAPG2-binding proteins were retrieved and analyzed using Gene Ontology (GO) analysis. Furthermore, the gene set enrichment analysis (GSEA) tool was then used to perform an analysis based on both HALLMARK and Kyoto Encyclopedia of Genes and Genomes (KEGG) pathway terms in order to identify enriched signaling pathways associated with NCAPG2.

### 2.6 NCAPG2 and drug response

The TIDE (http://tide.dfci.harvard.edu) and TISMO (http://tismo.cistrome.org) websites assessed the gene treatment responses and immunotherapy value of NCAPG2. The GSCA dataset (http://bioinfo.life.hust.edu.cn/GSCA/) was used to predict sensitive drugs based on NCAPG2 expression.

### 2.7 Statistical analysis

In this study, all statistical analyses were conducted using R software (version 3.6.4). A difference was considered statistically significant if P values < 0.05.

## 3 Results

### 3.1 Pan-cancer analysis of expression of NCAPG2

**Fig. 1:**
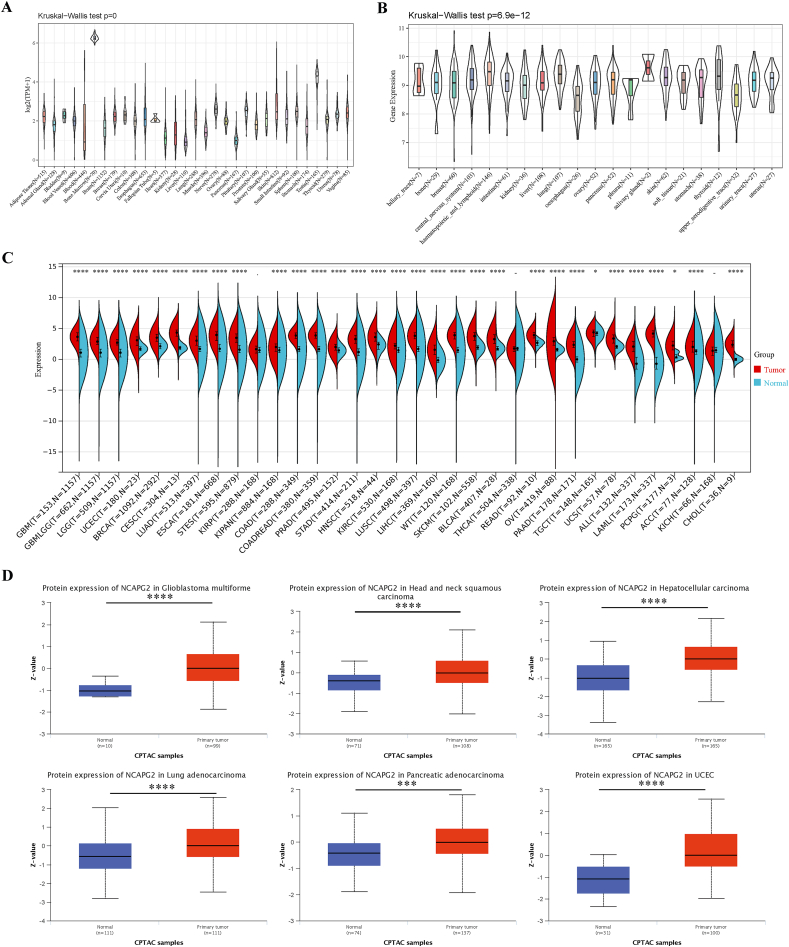
Expression and protein level of NCAPG2 in the normal and tumor samples. NCAPG2 levels in 31 normal tissues from the GTEx database(A). NCAPG2 levels in tumor cell lines from the CCLE database(B). NCAPG2 expression between tumor tissues analyzed by TCGA and GETx database(C). NCAPG2 protein expression level in normal tissue and Primary cancer were extracted and analyzed using CPTAC database(D). *p < 0.05; **p < 0.01; ***p < 0.001; ****p < 0.0001.

In this study, we downloaded RNA-seq data of NCAPG2 (ENSG00000146918) from three public databases. Additionally, full name of cohort in TCGA and TARGET databases were listed (Supplementary Table 1). The GTEx database was used to obtain information on NCAPG2 expression in normal tissues, which indicated that bone marrow, testis, and nerve tissues exhibit the highest levels of NCAPG2 (Fig. 1A). The CCLE database revealed significantly elevated expression of NCAPG2 in 38 tumor cell lines, with salivary gland, lung, and hematopoietic and lymphoid cell lines showing the highest levels of NCAPG2 enrichment (Fig. 1B). Data from the TCGA, GTEx, and TARGET databases indicated that NCAPG2 expression is significantly higher in tumor samples compared to that in normal samples in 31 tumor types: ACC, ALL, BRCA, BLCA, COAD/READ, CHOL, CESC, COAD, GBM, GBMLGG, ESCA, KIRC, KIPAN,HNSC, LUAD, LGG, LAML, LUSC, LIHC, PAAD, PRAD, READ, PCPG, STAD, STES, SKCM, OV, TGCT, UCS, UCEC, and WT (Fig. 1C).

In addition, a comparison of NCAPG2 protein expression using data from the CPTAC database demonstrated significantly higher total protein expression in primary HNSC, GBM, LUAD, LIHC, PAAD, and UCEC (Fig. 1D).

**Fig. 2:**
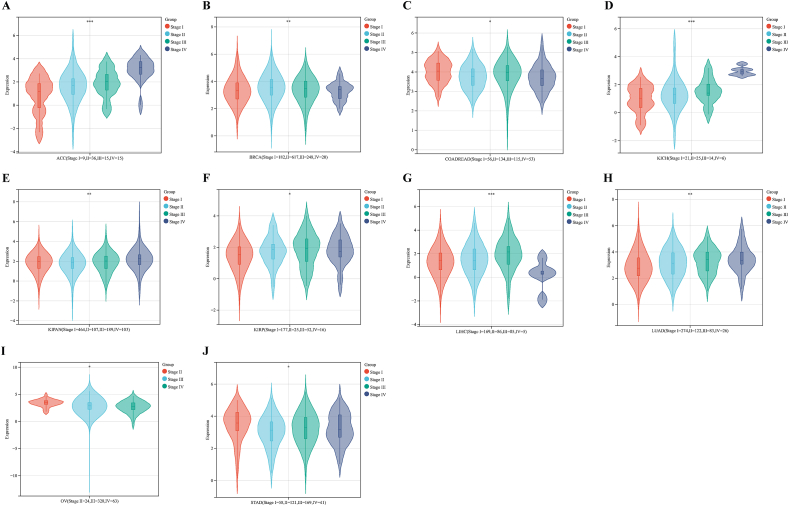
The correlation between NCAPG2 expression and the pathological stages of cancers. Main pathological stages (stage I, stage II, stage III, and stage IV) were investigated based on the TCGA database(A–J). *p < 0.05; **p < 0.01; ***p < 0.001.

We further assessed NCAPG2 expression at different tumor pathological stages and found that ACC, BRCA, COAD/READ, KIRP, KIPAN, KICH, LUAD, LIHC, OV, and STAD were significantly correlated with tumor stage (Fig. 2A–J).

### 3.2 Genetic alteration analysis

**Fig. 3:**
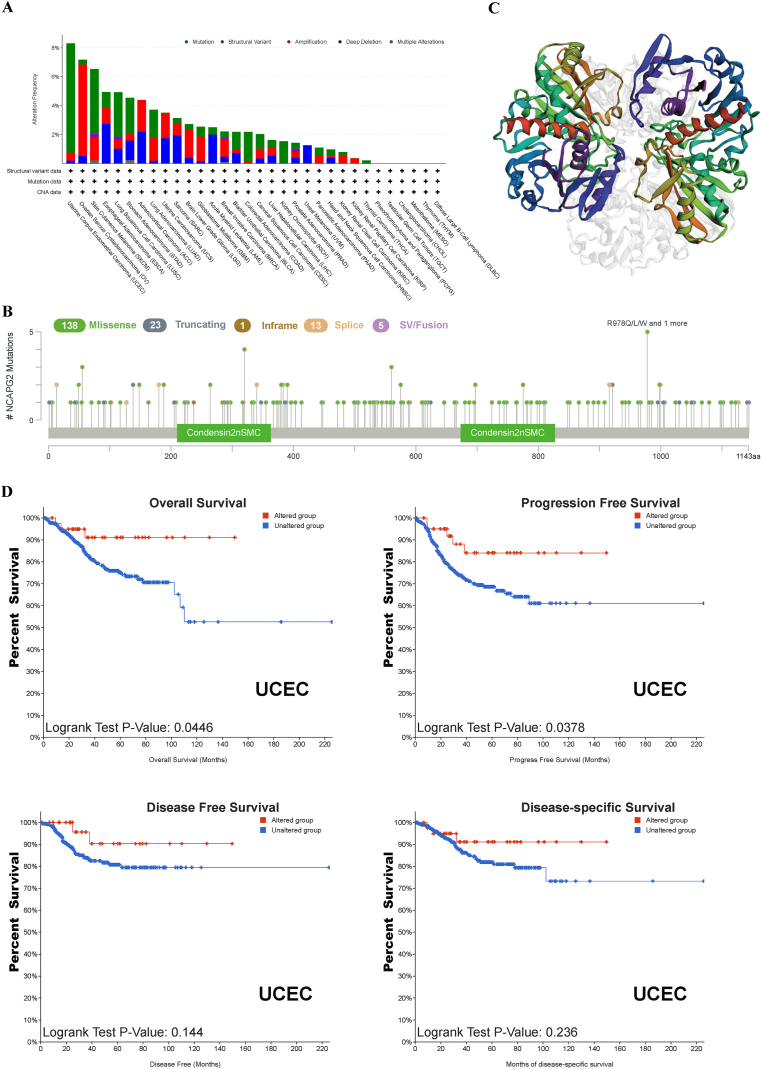
Mutation aspect of NCAPG2 in pan-cancer. Alteration frequency of NCAPG2 in pan-cancer (A). The entire mutation count of NCAPG2 from the TCGA database is based on the cBioPortal analysis (B). Mutation profile of NCAPG2 across protein domains (C). Relationship between NCAPG2 mutation status and prognosis using the cBioPortal tool (D).

Using the cBioportal dataset, we analyzed frequency changes and mutations in NCAPG2 across multiple cancers. Our findings indicate that UCEC, OV, SKCM, ESCA, LUSC, STAD, and ACC have high levels of mutation, with an alteration frequency of over 4% (Fig. 3A). To further investigate the mutation of NCAPG2, TCGA mutation data were plotted, and a total of 180 mutations were found, comprising 138 missense mutations, 23 truncating mutations, 1 in-frame mutations, 13 splices, and 5 fusions (Fig. 3B), as shown in the 3D protein structure (Fig. 3C). We conducted a comprehensive study to analyze the correlation between specific genetic alterations in NCAPG2 and their impact on clinical prognosis across multiple tumor types. The frequency of NCAPG2 alteration with “mutation” were highest in UCEC. Genetic alterations of NCAPG2 were correlated with improved PFS and OS in patients with UCEC; however, no such effect was noted in patients without NCAPG2 alterations. Moreover, no correlation was observed with DFS and DSS (Fig. 3D).

### 3.3 Prognostic value of NCAPG2

**Fig. 4:**
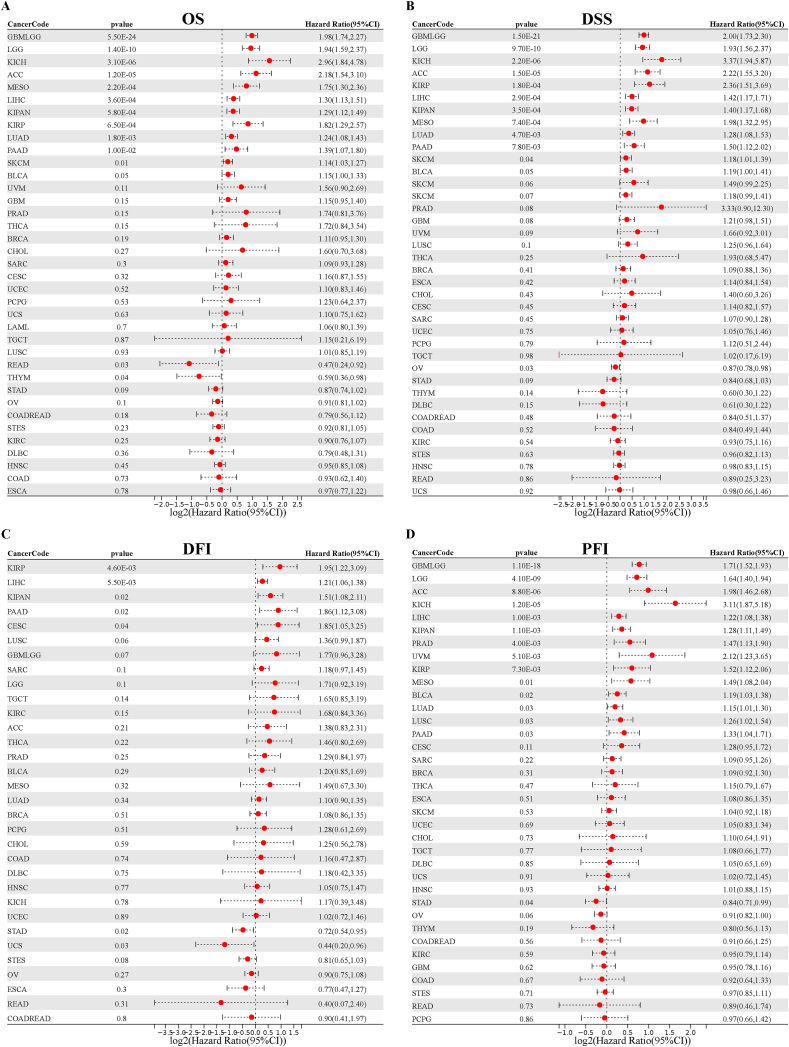
Univariate Cox regression analysis of NCAPG2 in TCGA pan-cancer. Survival analysis of NCAPG2 on OS (A),DSS (B), DFI(C),and PFI(D) in pan-cancer described by the forest map.

**Fig. 5:**
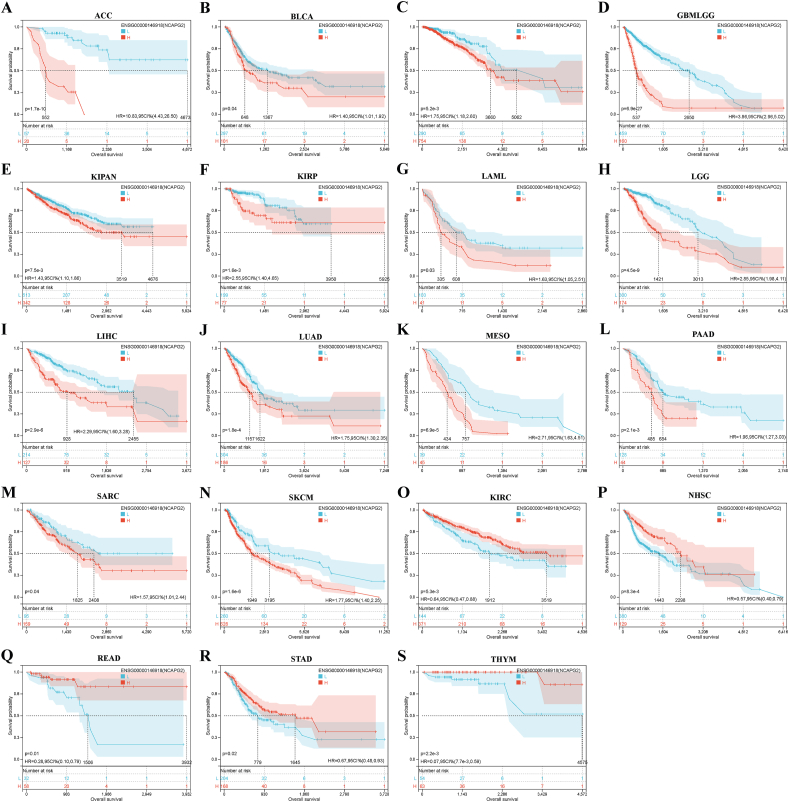
KM analysis of NCAPG2 on DSS in pan-cancer from the TCGA database(A-S).

We analyzed data from the TCGA database to investigate the correlation between NCAPG2 expression and cancer prognosis. The Cox regression analysis for overall survival indicated that NCAPG2 is a crucial risk factor for 11 cancer types (GBMLGG, LUAD, LIHC, LGG, KICH, KIPAN, KIRP, PAAD, MESO, ACC, and SKCM) and a significant protective factor for THYM and READ (Fig. 4A). From the results of KM survival curves, the OS results indicated that NCAPG2 serves as a significant risk factor for 14 cancer types (BRCA, BLCA, ACC, GBMLGG, KIPAN, KIRP, LIHC, LAML, LUAD, LGG, PAAD, MESO, SKCM, and SARC) (Fig. 5A–N) and as a significant protective factor for 5 cancer types (KIRC, HNSC, READ, STAD, and THYM) (Fig. 5O–S). DSS results indicated that NCAPG2 serves as a significant risk factor for 5 cancer types (GBMLGG, KIRP, KICH, KIPAN, LGG, LUAD, LIHC, MESO, PAAD, ACC, and SKCM) and as a protective agent for OV (Fig. 4B). From the results of KM survival curves, the DSS results suggested that NCAPG2 serves as a risk factor for 12 cancer types (ACC, BRCA, GBMLGG, LIHC, LGG, LUAD, KIPAN, KIRP, PAAD, PRAD, MESO, and SKCM) (Supplementary Fig. 1A-L) and as a protective factor for 5 cancer types (HNSC, KIRC, OV, THYM, and STAD) (Supplementary Fig. 1M-Q). DFI analysis indicated that NCAPG2 serves as a risk agent for 5 cancer types (CESC, KIPAN, KIRP, LIHC, and PAAD) and as a protective factor in STAD and UCS (Fig. 4C). The results from survival curves for DFI showed that NCAPG2 functions as a risk factor for 10 cancer types (CESC, KIPAN, KIRP, KIRC, PAAD, LUAD, LIHC, LUSC, SARC, and TGCT) (Supplementary Fig. 2A-K) and as a protective factor for 5 cancer types (ESCA, OV, STES, STAD, and UCS) (Supplementary Fig. 2L-O). The PFI analysis suggested that NCAPG2 acts as a protective factor for 14 cancer types (ACC, BLCA, KIRP, KIPAN, KICH, GBMLGG, LUAD, LIHC, LGG, LUSC, PAAD, PRAD, MESO, and UVM) and as a risk factor for STAD (Fig. 4D). The results from survival curves for PFI suggested that NCAPG2 serves as a risk factor for 16 cancer types (GBMLGG, KIRP, KICH, KIPAN, BLCA, ACC, PAAD, PRAD, LIHC, LGG, LUAD, LUSC, MESO, SKCM, UVM, and UCEC) (Supplementary Fig. 3A-L) and as a protective factor for 4 cancer types (KIRC, OV, THYM, and STAD) (Supplementary Fig. 3E-H).

### 3.4 Immune characteristics of NCAPG2 in TMEs

To identify the immunological features of NCAPG2 in TMEs, we utilized the ESTIMATE algorithm to analyze how NCAPG2 levels relate to immune scores, as well as estimated and stromal scores (Supplementary Figure 4-6). We found that cancers showing the strongest relationship between NCAPG2 levels and each score were WT, GBM, and LUSC for immune scores; WT, GBM, and TGCT for estimate scores; and NB, GBM, and STAD for stromal scores.

**Fig. 6:**
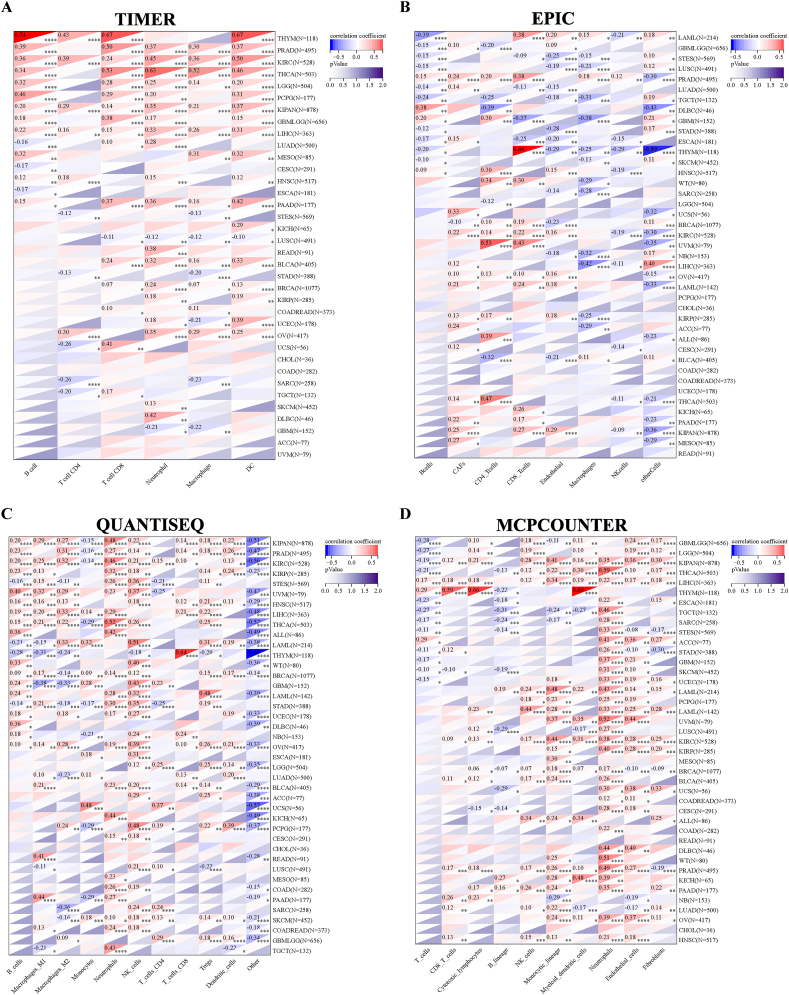
Correlation between NCAPG2 and immune infiltrates analyzed by the immunedeconv algorithm. Immune cell infiltration analyzed by the TIMER(A), EPIC(B), quanTIseq(C), and MCP-counter(D) algorithms. *p < 0.05, **p < 0.01, ***p < 0.001.

To further elucidate the association between NCAPG2 and infiltrated inflammatory cells in these cancers, we applied six of the latest algorithms (TIMER, EPIC, quanTIseq, MCP-counter, CIBERSORT, and xCell) to quantify immune cells (Fig. 6A–D and Supplementary Fig. 7A and B). Overall, our analysis of multiple cancer samples revealed a positive correlation between NCAPG2 and infiltrated inflammatory cells. In particular, increased NCAPG2 levels were found to have a significant correlated various infiltrating inflammatory cells, including macrophages, M2 macrophages, regulatory NK cells, cancer-associated fibroblasts (CAFs), T cells (Tregs), CD8^+^ T cells, CD4^+^ T cells, B cells, monocytes, neutrophils, and others, in BRCA, CESC, ESCA, DLBC, HNSC, GBMLGG, GBM, KIRC, KIRP, KIPAN, KICH, LIHC, LUAD, LGG, LUSC, OV, MESO, STAD, SARC, STES, SKCM, PRAD, PAAD, THYM, TGCT, and THCA (P < 0.05).

We delved deeper into the correlation between NCAPG2 and MSI and TMB. We found a positive link between NCAPG2 and MSI in ACC, GBM, LUSC, LUAD, SARC, STAD, and STES. However, we discovered that NCAPG2 was negatively correlated with MSI in other cancers, such as DLBC, GBMLGG, HNSC, and THCA (Supplementary Figure 8A). The levels of NCAPG2 were found to be positively correlated with TMB in several types of cancer, including ACC, LUAD, PRAD, STAD, STES, and KICH (Supplementary Figure 8B). We then investigated the association between NCAPG2 and inhibitory and stimulatory immune checkpoints expression (Supplementary Figure 8C). Our findings revealed a strong positive correlation between NCAPG2 and the immune checkpoints in several cancer types, including BLCA, GBMLGG, HNSC, KIRC, KIPAN, LIHC, OV, PRAD, PAAD, and UVM (P < 0.05).

### 3.5 PPI network and enrichment analysis of NCAPG2

**Fig. 7:**
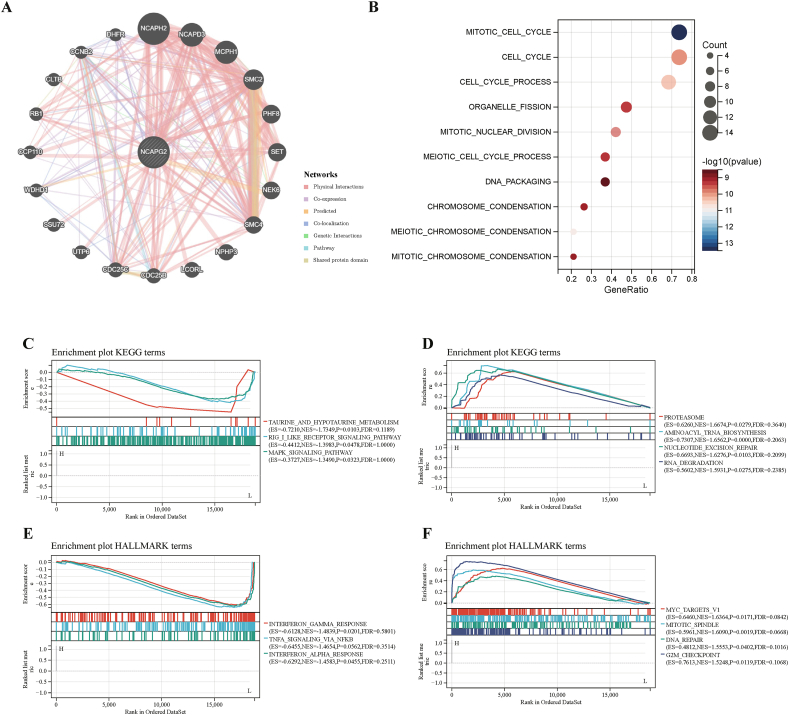
PPI network and Functional analysis for NCAPG2. PPI network for NCAPG2 was constructed in GeneMANIA(A). Enrichment pathways analysis of NCAPG2 from the GSVA algorithm(B). Top three negative (C) and top four positive (D) enriched pathways based on the KEGG terms. Top three negative (E) and top four positive (F) enriched pathways based on the HALLMARK terms.

Next, to further understand the potential involvement of NCAPG2 in carcinogenesis, we constructed a PPI network for NCAPG2 by GeneMANIA dataset. NCAPG2 showed strong physical interactions with MCPH1, NCAPD3, NCAPG2, NCAPH2, and SMC2 (Fig. 7A). Enrichment analysis of the PPI network revealed that NCAPG2 was primarily involved in the cell cycle, cell cycle process, and mitotic cell cycle among GO biological processes annotation terms (Fig. 7B). The top four pathways that showed negative enrichment using the KEGG gene set were taurine and hypotaurine metabolism, allograft rejection, MAPK signaling, and RIG-1-like receptor signaling (Fig. 7C). Additionally, the top four with the highest positive scores were proteasome, aminoacyl tRNA biosynthesis, nucleotide excision repair, and RNA degradation (Fig. 7D). According to the HALLMARK gene set, the top four pathways with the most negative scores were TNFA signaling via NFKB, interferon-gamma response, interferon alpha response, and KRAS signaling DN (Fig. 7E). On the other hand, the top four pathways with the highest positive scores were DNA repair, MYC targets v1, mitotic spindle, and G2M checkpoint (Fig. 7F).

### 3.6 Immunotherapy value and sensitive drug prediction

**Fig. 8:**
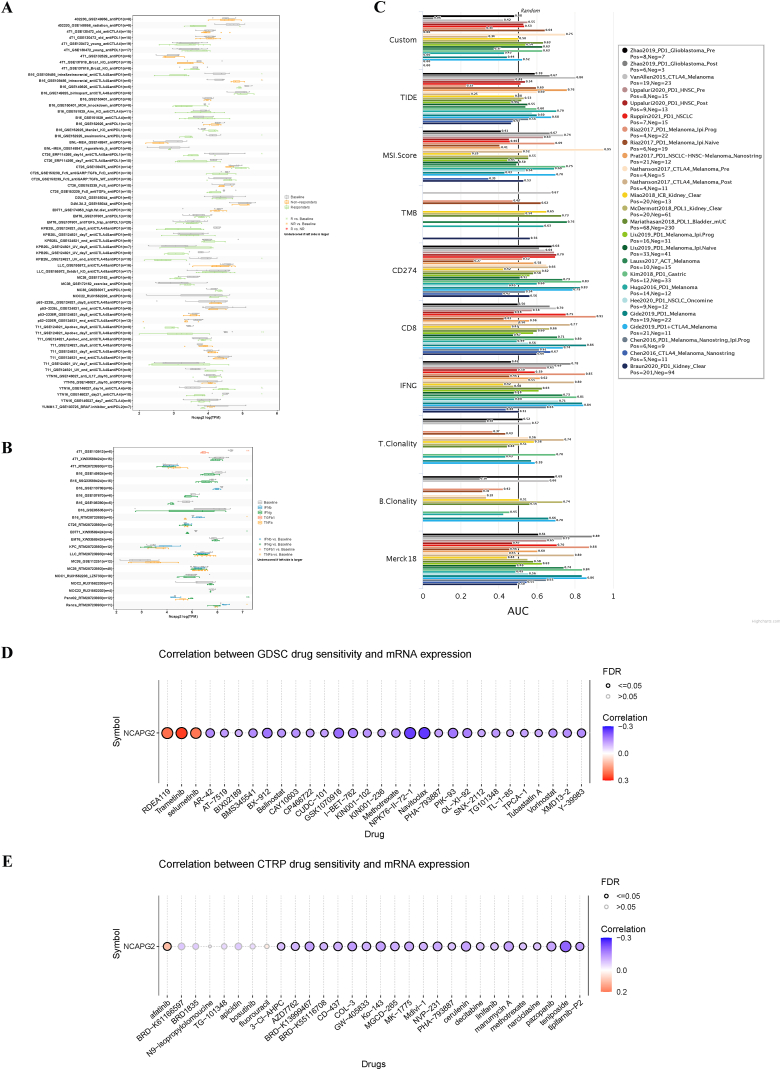
Immunotherapeutic value of NCAPG2 in pan-cancer. Immunotherapy response of NCAPG2 in murine immunotherapy cohorts based on in vivo studies (A) and in vitro studies (B).The biomarker relevance of NCAPG2 in immunotherapy cohorts (C). Predictive drugs based on NCAPG2 expression in pan-cancer from the GDSC (D) and CTRP (E) datasets.

To completely investigate the immune aspect of NCAPG2, we used NCAPG2 expression to explore the response to immunotherapy and drug sensitivity in multiple tumors. Notably, in eight murine immunotherapy cohorts (Fig. 8A) and seven Turin cytokine treatment cohorts NCAPG2 expression levels could accurately predict immunotherapy response (Fig. 8B). We calculated the biomarker relevant to NCAPG2 with standardized biomarkers in human immunotherapy cohorts, based on their predictive ability for OS and response outcomes. NCAPG2 showed an AUC above 0.5 in 10 out of the 25 immunotherapy cohorts analyzed (Fig. 8C), suggesting better predictability compared to B. Clonality, T. Clonality, and TMB, which had AUC values above 0.5 in 7 cohorts. NCAPG2 showed a lower predictive value compared to CD8, CD274, TIDE, MSI score, and IFNG, which showed AUC values above 0.5 in 18, 21, 18, 13, and 17 immunotherapy cohorts, respectively. We used public databases to predict molecules and drugs for various types of tumors based on NCAPG2 levels. Positive correlations indicated gene resistance to the drug, whereas negative correlations indicated susceptibility. According to the Genomics of Drug Sensitivity in Cancer (GDSC) database, there is a negative association between NCAPG2 levels and drug sensitivity. Specifically, Navitoclax, NPK76-II-72-1, and GSK1070916 are the top three drugs that are negatively correlated with NCAPG2 (Supplementary Table 2 and Fig. 8D). In contrast, Selumetinib, RDEA119, and Trametinib, which are inhibitors of MEK1/MEK2, were found to have the strongest positive correlation with NCAPG2 (Supplementary Table 3 and Fig. 8D). According to the Cancer Therapeutics Response Portal (CTRP) dataset, there is a negative correlation between NCAPG2 and drug sensitivity. Specifically, NSC19630, BIX-01294, and mitomycin were found to be the top three drugs negatively correlated with NCAPG2 expression (Supplementary Table 2 and Fig. 8E).

## 4 Discussion

NCAPG2 belongs to the sorting nexin protein family, responsible for transport and protein sorting as well as cell mitosis [12,13]. Previous studies have demonstrated that NCAPG2 is closely linked to proliferation, invasion, and migration [[8], [9], [10]]. Our study performed a pan-cancer analysis to evaluate expression levels and genetic alterations of NCAPG2 and found that NCAPG2 were elevated in tumor samples compared to normal samples across various types of cancer, indicating that NCAPG2 may serve as a critical oncogene for cancer development and progression. Furthermore, we systematically collected and integrated protein data, as well as clinical features, using the UALCAN database and found that NCAPG2 was associated with clinical stage and prognosis in various cancers. The results of our study suggest that NCAPG2 could be a valuable diagnostic and prognostic biomarker in multiple cancers.

Significant advancements and numerous breakthroughs in cancer immunotherapy have been made in recent decades, resulting in improved clinical outcomes for patients with various types of cancer [14]. Immunotherapy for tumors has substantially enhanced both the quality of life and survival rates [15]. Nevertheless, the immunosuppressive status of the TMEs can facilitate tumor progression, invasion, and therapy resistance [16]. Immune checkpoints promote the evasion of immune surveillance by tumor cells. Owing to the upregulation of immune checkpoints in tumors, the killing effect of T cells could be blunted. Immune checkpoint inhibitors are among the most promising immunotherapies. Advancements in high-throughput sequencing have led to the discovery of several new immune checkpoints [17]. We then investigated the potential of NCAPG2 as a novel immunotherapy target in TMEs and found that high levels of NCAPG2 is closely associated with estimate, stromal, and immune scores. We observed a positive correlation between NCAPG2 expression and MSI as well as TMB. Furthermore, we conducted an in-depth analysis of NCAPG2 and other immune checkpoints. Results demonstrate that NCAPG2 is positively correlated with several immune checkpoints, including CD276, VEGFA, LAG3, VEGFB, IL13, HMGB1, CX3CL1, ICOSLG, TNFSF9, and IFNA1. The analyses conducted demonstrated the potential of NCAPG2 as an immune microenvironment indicator. Moreover, NCAPG2 has shown promise as a predictive indicator for response to immunotherapy.

Inflammatory cells in TMEs can either promote or inhibit tumor growth and the effectiveness of anti-tumor immunotherapy. Understanding their role is crucial for developing effective cancer treatments [18]. T lymphocytes are indispensable components of the adaptive immune response to combat tumors. Tregs are essential components of immunosuppressive microenvironments, as they inhibit the differentiation and activation of CD4^+^ helper T cells and CD8^+^ cytotoxic T cells [19]. During the immune response against tumors, Tregs secrete cytokines including IL-35, TGF-β, and IL-10, which suppress anti-tumor immunity and facilitate the progression and formation of cancers [20]. As a major component of TMEs, tumor-associated macrophages (TAMs) can regulate inflammation. Furthermore, they can either facilitate, inhibit, or initiate tumor development by secreting cytokines and modulating the functions of other immune cells [21,22]. Mounting evidence has demonstrated that tumor-associated macrophages, especially the M2 subtype, play a crucial role in promoting an immunosuppressive TMEs by facilitating the recruitment of Tregs and hampering the differentiation and function of T cells [23,24]. Tumor-associated neutrophils can have opposite effects on tumor immunity depending on their subtype, which can be either anti-tumorigenic or pro-tumorigenic, according to several studies [25]. CAFs are essential components of the tumor mesenchyme in non-neoplastic cells. They are essential for promoting tumor progression and metastasis by supporting cancer cell growth, invasion, and survival. CAFs interact with tumor cells through various complex mechanisms, including the secretion of extracellular matrix (ECM), growth factors，and cytokines [26]. According to previous non-small-cell lung cancer research, NCAPG2 is strongly correlated between NCAPG2 and immune infiltration levels [27]. Our research investigated the correlation between NCAPG2 and the infiltration of inflammatory cells within TMEs. NCAPG2 maintained be closely associated with various inflammatory cells in most cancers, including macrophages, M2 macrophages, T cells, Tregs, CAFs, monocytes, neutrophils, and NK cells. These results demonstrate an association between NCAPG2 and tumor and stromal cells in TMEs. NCAPG2 has been shown to participate in various immune-related pathways, including the regulation of mast cell, T cell, fibroblast, and macrophage proliferation, activation, and migration. Overall, these findings suggest that NCAPG2 is associated with an immunosuppressive microenvironment in cancers.

It is becoming increasingly common to utilize computational models and public datasets to find the best personalized therapeutic drugs [28]. Therefore, in the final aspect of this study, we utilized publicly available databases to assess the predictive significance of NCAPG2 by computing its biomarker relevance across 25 immunotherapy cohorts. We found that NCAPG2 alone had predictive value in 10 immunotherapy cohorts and in 7 cohorts, NCAPG2 outperformed B. Clonality, T. Clonality, and TMB in terms of predictive value. However, in over 10 immunotherapy cohorts, the predictive value of NCAPG2 was found to be lower compared to biomarkers such as CD274, TIDE, MSI score, CD8, and IFNG. More importantly, our findings indicate that NCAPG2 has predictive value for immunotherapy response in both in vivo and in vitro biological experiments. In addition, we predicted several candidates targeted small-molecule drugs using two public databases. This provides a novel approach for expanding the therapeutic options of these targeted small-molecule drugs and for developing new agents that specifically target NCAPG2.

To sum up, this study elucidated the prognostic and immunotherapy value of NCAPG2 using pan-cancer analysis. However, it should be pointed out that our study was limited by the fact that it was a pure computational result, and lacked biological experiments to validate. Therefore, further experiments are necessary to confirm our results. Overall, future research on NCAPG2 expression and its impact on TMEs could potentially lead to the development of more effective and personalized immunotherapy-based anti-cancer strategies.

## Data Availability Statement

The datasets utilized in this study are available in online repositories, while the original contributions can be accessed through the article or supplementary material.

## Funding

None.

## Declaration of competing interest

The authors declare that they have no known competing financial interests or personal relationships that could have appeared to influence the work reported in this paper.
