# Supplementary material for: Pan-cancer analysis of the immune aspects and prognostic value of NCAPG2
Source: Heliyon. 2023 Jul 13;9(7):e18051. doi: 10.1016/j.heliyon.2023.e18051 (PMC10368848; doi:10.1016/j.heliyon.2023.e18051)
Supplement: Multimedia component 1 [file mmc1.docx]

**Supplementary Figures**


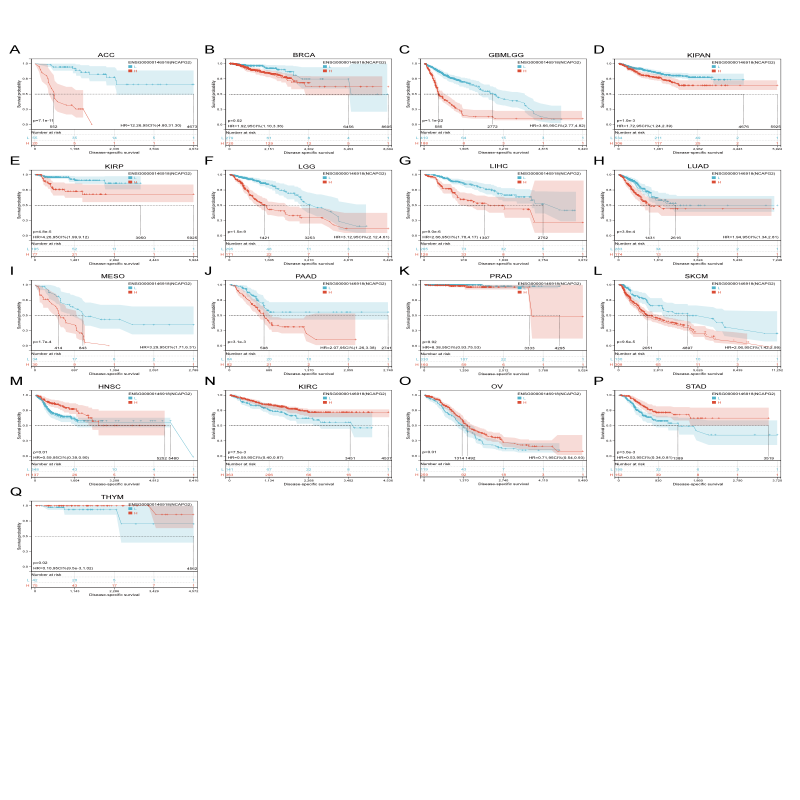


Supplementary Figure 1 | KM analysis of NCAPG2 on DSS in pan-cancer from the TCGA database(A-Q).


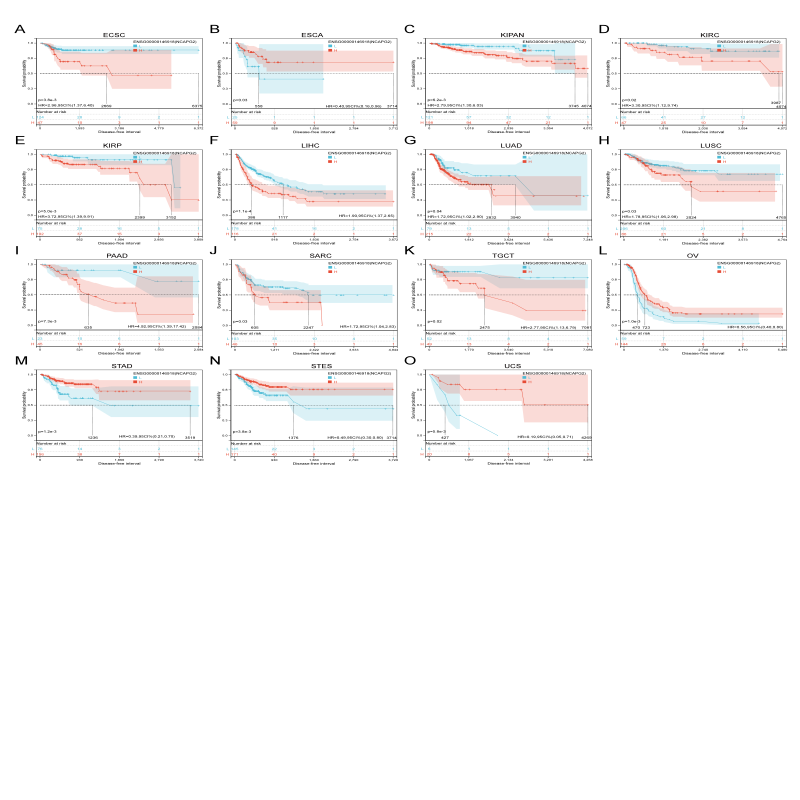


Supplementary Figure 2 | KM analysis of NCAPG2 on DFI in pan-cancer from the TCGA database(A-O).


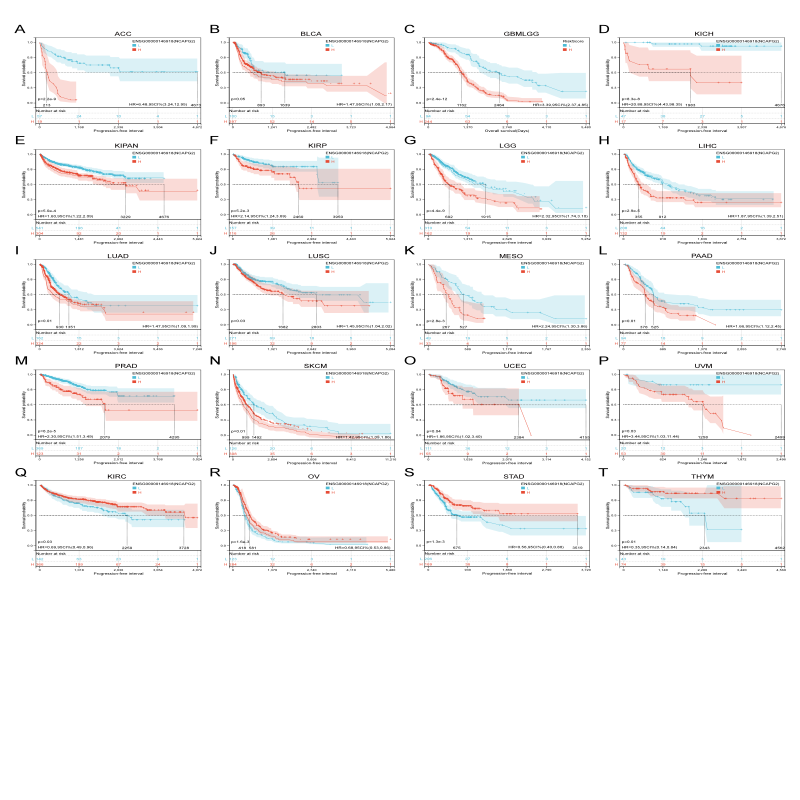


Supplementary Figure 3 | KM analysis of NCAPG2 on PFI in pan-cancer from the TCGA database(A-H).


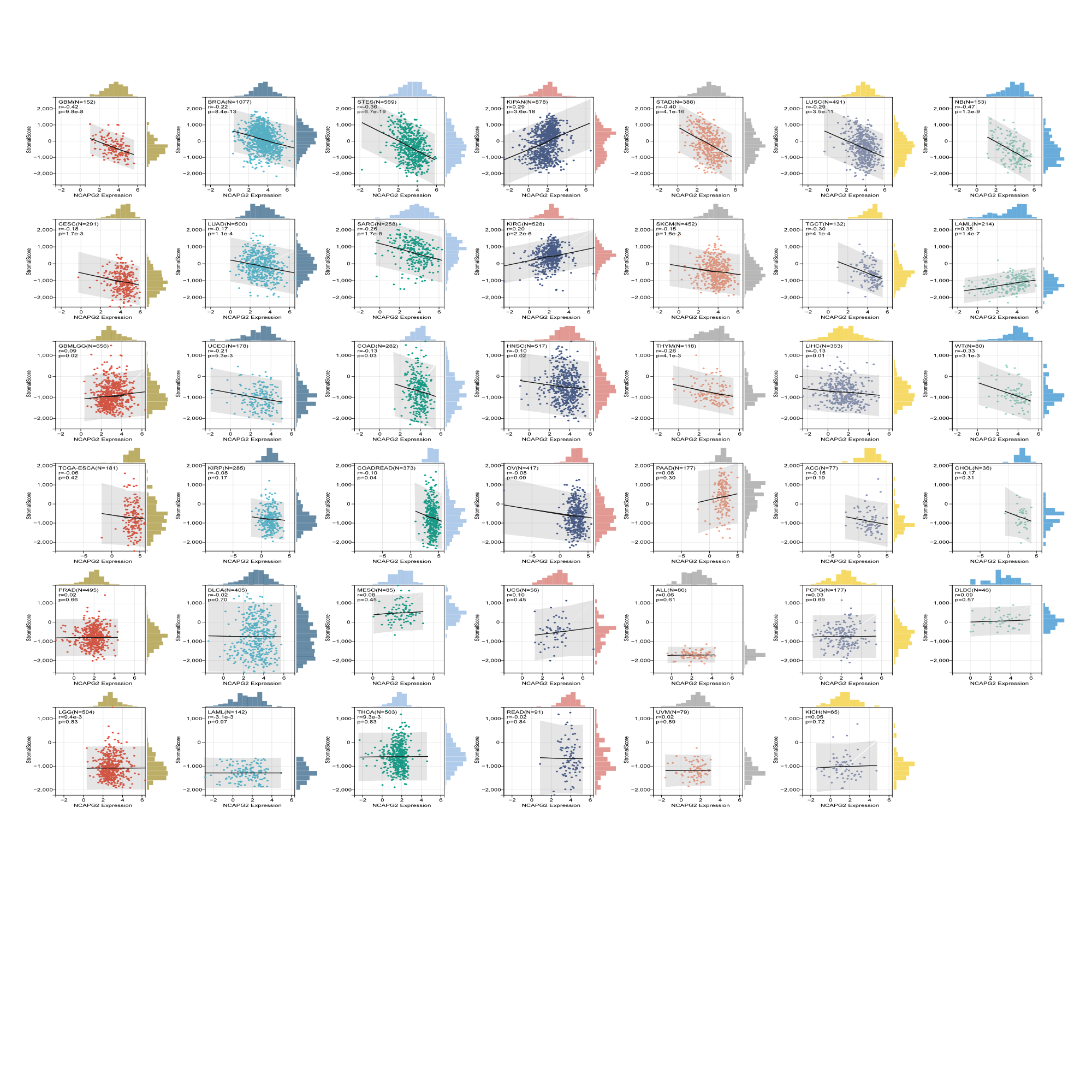


Supplementary Figure 4 | Relationship between NCAPG2 expression and the immune scores.


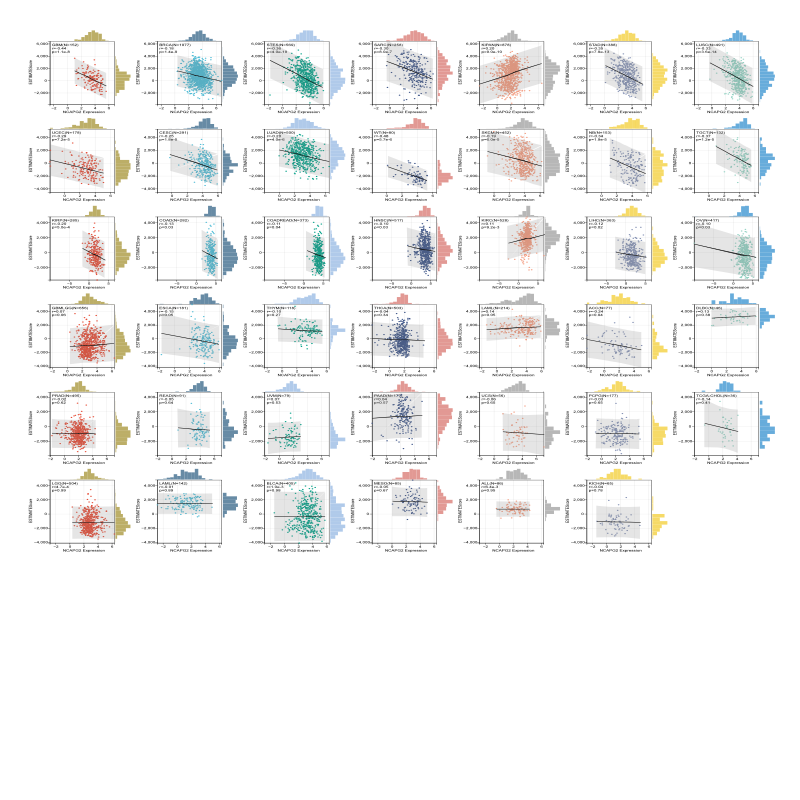


Supplementary Figure 5 | Relationship between NCAPG2 expression and the estimate scores.


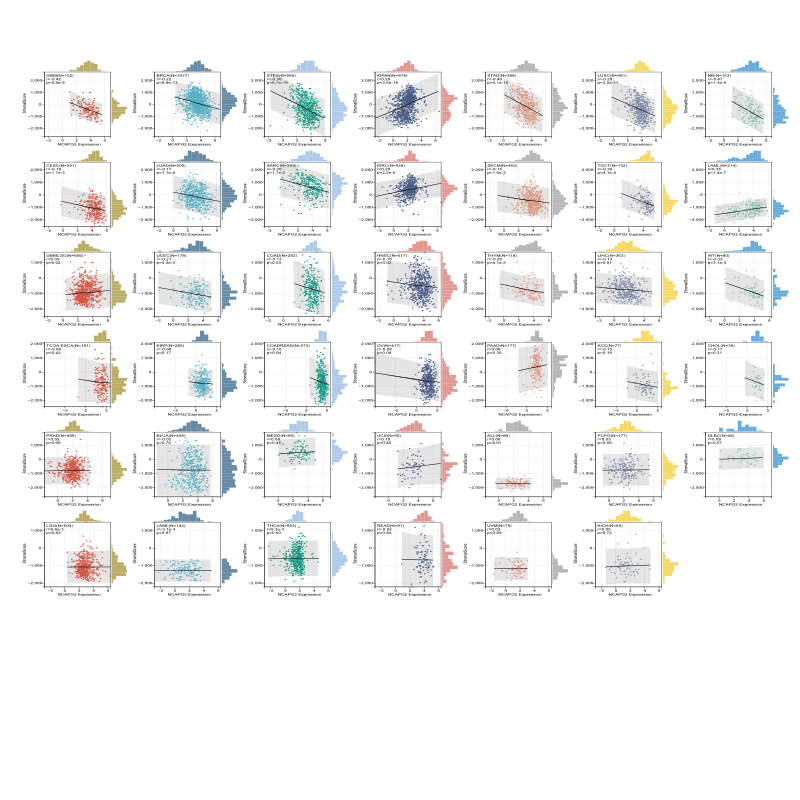


Supplementary Figure 6 | Relationship between NCAPG2 expression and the stromal scores.


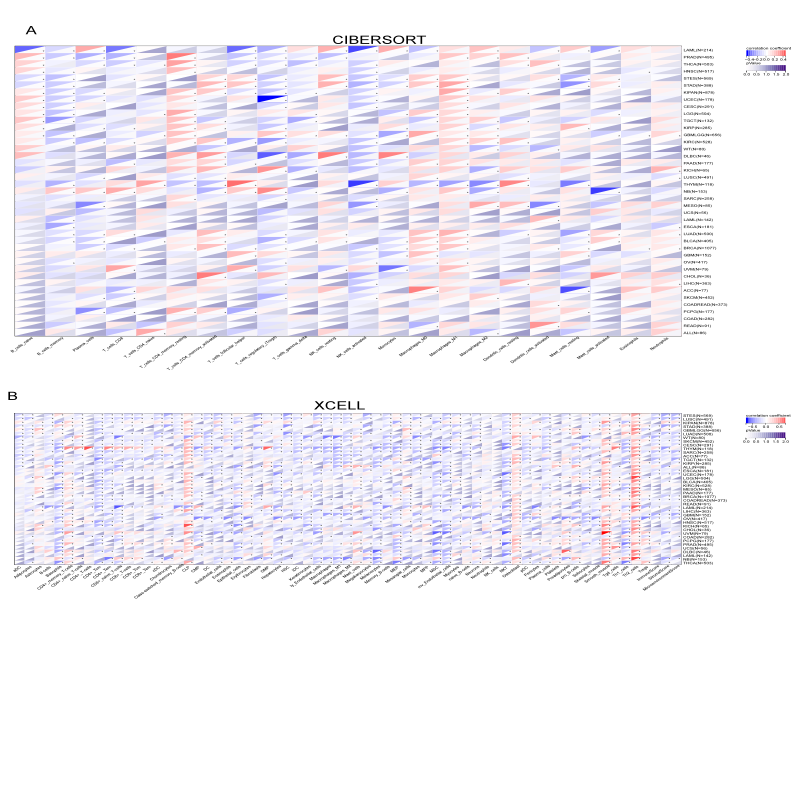


Supplementary Figure 7 | Immune cell infiltration was analyzed by the CIBERSORT (A) and xCell (B) algorithms. *p< 0.05.


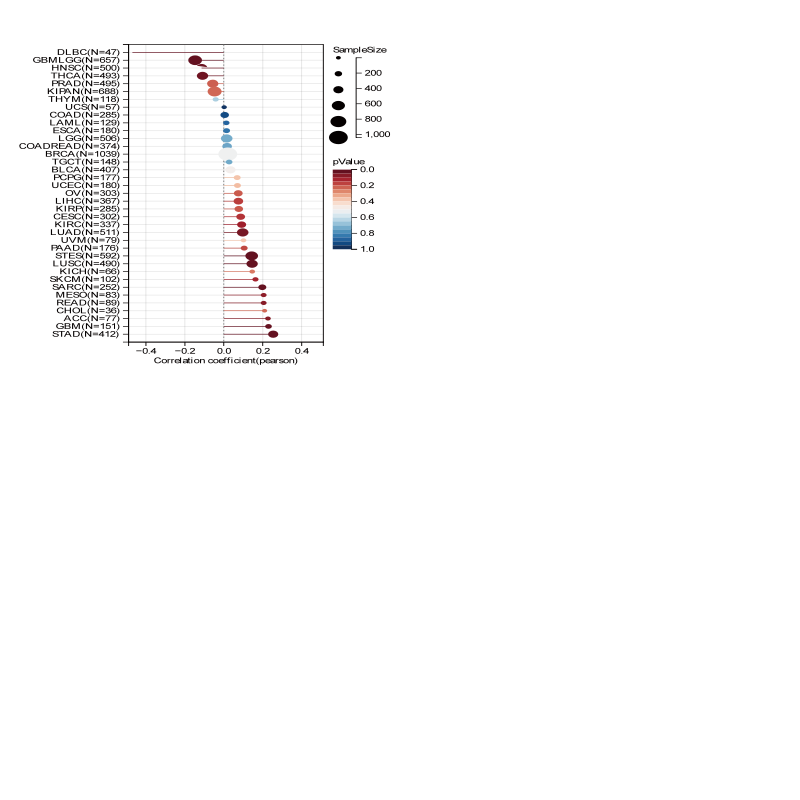


Supplementary Figure 8 | Relationship between NCAPG2 expression and MSI, TMB, and immune checkpoints in pan-cancer. Relationship between NCAPG2 expression and MSI displayed by the forest plot(A). Relationship between NCAPG2 expression and TMB displayed by the forest plot (B). Correlation between NCAPG2 and immune checkpoints (C). *p< 0.05.
